# MSCs promote the efferocytosis of large peritoneal macrophages to eliminate ferroptotic monocytes/macrophages in the injured endometria

**DOI:** 10.1186/s13287-024-03742-z

**Published:** 2024-05-01

**Authors:** Jiali Wang, Jingman Li, Lijie Yin, Xiuzhu Wang, Yue Dong, Guangfeng Zhao, Sunan Shen, Yayi Hou

**Affiliations:** 1 The State Key Laboratory of Pharmaceutical Biotechnology, Division of Immunology, Medical School, Nanjing University, No. 22 Hankou Rd., Gulou District, Nanjing, Jiangsu, 210093 People’s Republic of China; 2 Department of Obstetrics and Gynecology, Affiliated Hospital of Medical School, Nanjing Drum Tower Hospital, Nanjing University, Nanjing, China; 3 Jiangsu Key Laboratory of Molecular Medicine, Medical School, Nanjing University, Nanjing, 210093 China

**Keywords:** Large peritoneal macrophages, Ferroptosis, Efferocytosis, Mesenchymal stem cells, Endometrial injury

## Abstract

**Background:**

Endometria are one of the important components of the uterus, which is located in the peritoneal cavity. Endometrial injury usually leads to intrauterine adhesions (IUA), accompanied by inflammation and cell death. We previously reported that both the endometrial ferroptosis was increased and monocytes/macrophages were involved in endometrial injury of IUA. Large peritoneal macrophages (LPMs) are recently reported to migrate into the injured tissues and phagocytose dead cells to repair the tissues. We previously demonstrated that mesenchymal stromal cells (MSCs) had made excellent progress in the repair of endometrial injury. However, it is unclear whether MSCs regulate the LPM efferocytosis against ferroptotic monocytes/macrophages in the injured endometria.

**Methods:**

Here, endometrial injury in IUA mouse model was conducted by uterine curettage and LPS injection surgery and the samples were collected at different times to detect the changes of LPMs and ferroptotic monocytes/macrophages. We conducted LPMs depletion assay in vivo and LPMs and Erastin-induced ferroptotic THP-1 cells coculture systems in vitro to detect the LPM efferocytosis against ferroptotic monocytes/macrophages. The IUA model was treated with MSCs, and their effects on LPMs and endometrial repair were analyzed. Flow cytometry, western blotting, quantitative real-time PCR, immunohistochemical analysis, ELISA, and RNA-sequencing were performed.

**Results:**

We found that LPMs migrated to the injured uteri in response to the damage in early phase (3 h), and sustained to a later stage (7 days). Astonishingly, we found that ferroptotic monocytes/macrophages were significantly increased in the injured uteri since 12 h after injury. Moreover, LPMs cocultured with Erastin-induced ferroptotic THP-1 cells in vitro, efferocytosis of LPMs against ferroptotic monocytes/macrophages was emerged. The mRNA expression profiles revealed that LPM efferocytosis against ferroptotic monocytes/macrophages was an induction of glycolysis program and depended on the PPARγ-HK2 pathway. Importantly, we validated that MSCs promoted the efferocytic capability and migration of LPMs to the injured uteri via secreting stanniocalcin-1 (STC-1).

**Conclusion:**

The data collectively demonstrated first the roles of LPMs via removal of ferroptotic monocytes/macrophages and provided a novel mechanism of MSCs in repairing the endometrial injury.

**Graphical Abstract:**

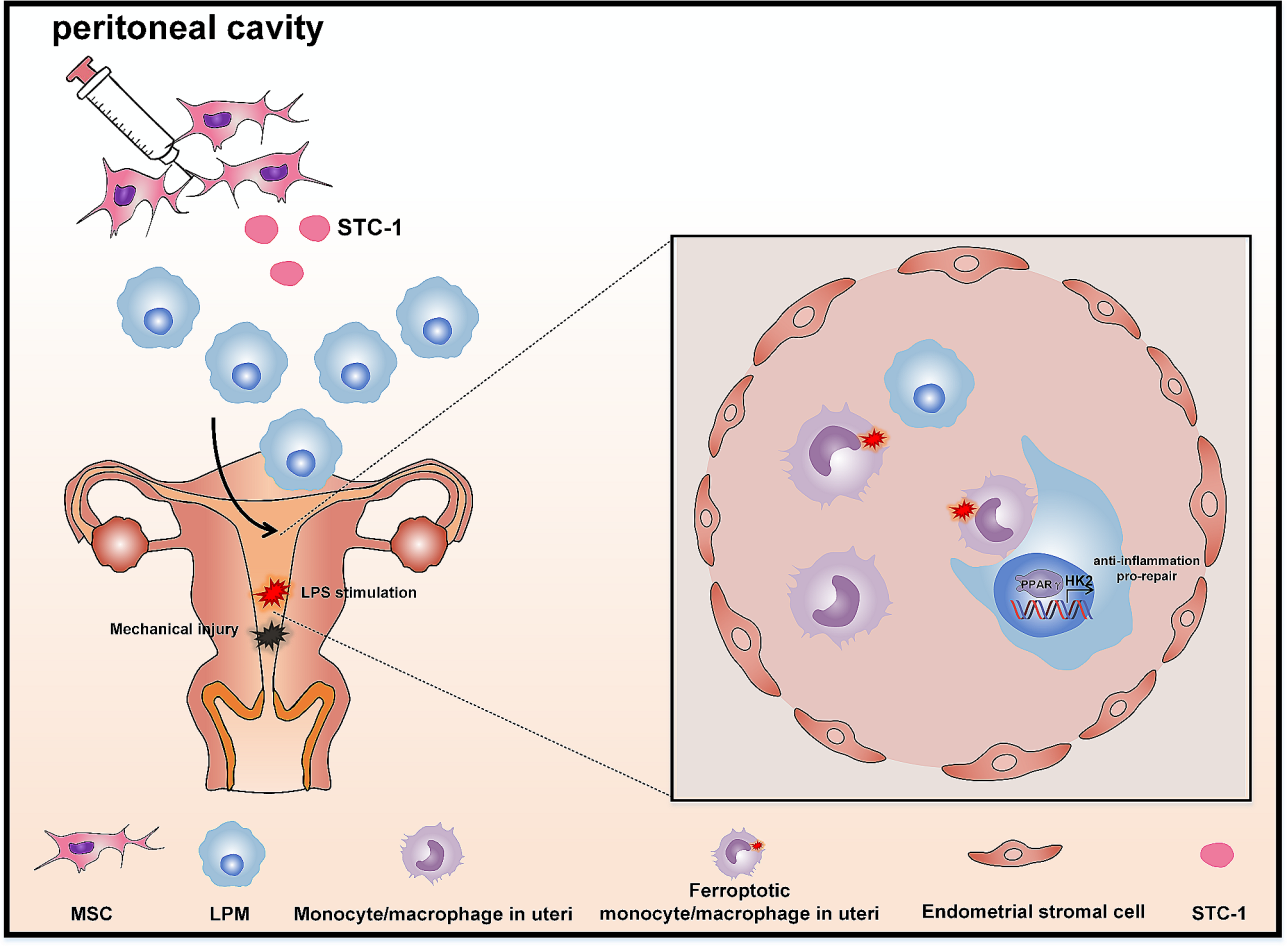

**Supplementary Information:**

The online version contains supplementary material available at 10.1186/s13287-024-03742-z.

## Background

Endometrial injury due to repeated intrauterine operations usually lead to intrauterine adhesions (IUA), which is a common syndrome of uterine infertility patients, accompanied by inflammation and cell death [1, 2]. Moderate-to-severe IUA may cause infertility of women within reproductive age. Endometrium is one of the important components of the uterus, which located in the peritoneal cavity. Current studies have reported that under physiological conditions, there are two kinds of macrophage populations in the peritoneal cavity of mammals, namely CD11b^+^ F4/80^hi^ CD102^+^, large peritoneal macrophages (LPMs) and CD11b^+^ F4/80^lo^ MHCII^+^, small peritoneal macrophages (SPMs) [3–6]. Research has established that LPMs not only fulfill peritoneal homeostatic functions, but are also involved in repair of tissue damage caused by inflammation and infection. In response to injury in the peritoneal cavity such as liver [7], or the pericardial cavity such as heart [8], LPMs can rapidly migrate into afflicted tissues and fulfill an essential role in the rapid clearance of the mass of cellular death and debris, promote an anti-inflammatory phenotype and help inflammation resolution [9]. However, whether LPMs can migrate into the uteri to phagocytose dead cells effectively under endometrial injury remains unknown.

We and other authors previously reported that ferroptosis occurred in the injured endometria to promote endometrial fibrosis in IUA [10, 11]. Ferroptosis is a novel form of oxidative cell death, which is regulated by glutathione peroxidase 4 (GPX4) and caused by iron-dependent oxidative damage [12, 13]. Moreover, ferroptosis in macrophages has been reported in several inflammation-related diseases [14–16]. For example, STING-regulated macrophage ferroptosis promoted sepsis-induced intestinal injury [14]. Phagocytosis of JAK2VF RBCs by plaque macrophages promoted ferroptosis that exacerbated atherosclerosis [15]. ACSL4 inhibition prevented macrophage ferroptosis and alleviated fibrosis in bleomycin-induced systemic sclerosis model [16]. Our previous studies have revealed that phenotypically and functionally distinct macrophages such as CX3CR1^+^ monocytes/macrophages or CD301^+^ macrophages promoted the differentiation of endometrial stromal cells into myofibroblasts and resulted in extracellular matrix accumulation, which drove inflammation and endometrial fibrosis in IUA [17, 18]. But it remains unknown whether ferroptosis occurred in monocytes/macrophages which participated in the injured endometria.

We also previously demonstrated that mesenchymal stromal cells (MSCs) had made excellent progress in the repair of endometrial injury [19–21]. The studies revealed that MSCs directly interacted with various types of immune cells to maintain homeostasis in the injured tissues [22, 23]. However, to date, it is not reported on the regulation of MSCs on the efferocytosis and migration of LPMs in the injured endometria.

In the present study, we demonstrated that LPMs migrated to the uteri in response to the damage in early phase (3 h), and sustained to a later stage (7 days). Meanwhile, the proportion of ferroptotic monocytes/macrophages was significantly increased in the injured uteri since 12 h after injury. Through LPMs depletion assay in vivo and coculture systems in vitro, we found that LPM efferocytosis against ferroptotic monocytes/macrophages, and this process appeared an induction of glycolysis program and depended on the PPARγ-HK2 pathway. Furthermore, MSCs promoted the efferocytosis of LPMs to repair the injured uteri through secreting stanniocalcin-1 (STC-1). Collectively, our research demonstrated the essential functions of LPMs for endometrial repair and provided a novel mechanism of MSCs in repairing the injured endometria.

## Methods

### Reagents

Erastin (Catalog # HY-15,763), Cytochalasin D (Catalog # HY-N6682), 2-Deoxy-D-glucose (Catalog # HY-13,966), Rosiglitazone (Catalog # HY-17,386), T0070907 (Catalog # HY-13,202), and Bafilomycin A1 (Catalog # HY-100,558) were purchased from MedChem Express (Monmouth Junction, NJ, USA). MDA (Catalog # ab243066) was purchased from Abcam (Cambridge, MA, USA), ROS (Catalog # S0033S) was purchased from Beyotime (Shanghai, China), Recombinant human STC-1 (Catalog # 9400-S0-050) was purchased from R&D (Boston, MO, USA). Clodronate liposome was purchased from Liposoma B.V (Vrije University, Netherlands). LPS derived from Escherichia coli (Catalog # 0111: B4) was purchased from Sigma-Aldrich (St. Louis, MO, USA).

### Cell cultures

THP-1 cells were cultured in 1640 (Gibco, Grand Island, NY, USA) containing 10% FBS (Gibco, Grand Island, NY, USA), 1% penicillin and streptomycin (100 µg/mL; Gibco, Grand Island, NY, USA), at 37 °C in a humidified atmosphere with 5% CO2.

Mouse peritoneal macrophages were obtained from 8- to 10-week-old female Balb/c mice as previously described [24]. Peritoneal macrophages were cultured in DMEM (Gibco, Grand Island, NY, USA) containing 10% FBS (Gibco, Grand Island, NY, USA), 1% penicillin and streptomycin (100 µg/mL; Gibco, Grand Island, NY, USA), at 37 °C in a humidified atmosphere with 5% CO2.

Human umbilical cord mesenchymal stem cells (hUC-MSCs) were purchased from the Nanjing Drum Tower Clinical Stem Cell Center. MSCs were cultured in Dulbecco’s modified Eagle’s medium/F12 (Gibco, Grand Island, NY, USA) supplemented with 1% penicillin and streptomycin (100 µg/mL; Gibco, Grand Island, NY, USA), and 10% FBS (Gibco, Australia origin). MSCs between passages 3 and 5 were used in subsequent experiments.

### In vitro efferocytosis assays

THP-1 cells were treated with Erastin (80 µM) for 24 h and stained with CFSE (2.5 µg / 1 × 10^7^ cells) (Catalog # 65-0850-85; Invitrogen, Thermo Fisher Scientific, Inc., Waltham, MA, USA) for 10 min. Stained cells were collected by centrifugation at 300×g for 5 min. Peritoneal macrophages were incubated with CSFE-stained ferroptotic THP-1 cells at a ratio of 3:1 for 4 h. The MFI of CFSE in LPMs containing the ingested ferroptotic THP-1 cells was determined by flow cytometry or immunofluorescence assay.

### Immunofluorescence assay

LPMs were incubated with CSFE-stained ferroptotic THP-1 cells. Cells were fixed in 4% paraformaldehyde and incubated in blocking buffer (5% BSA in PBS) for 1 h at room temperature. Cells were then incubated with rat anti-mouse CD102 (Catalog # 10121-2-AP; Proteintech, Wuhan, China) overnight at 4 ℃. After rinsing thrice with PBS, cells were incubated with Alexa Fluor 647 conjugated goat anti-rat IgG secondary antibody (Catalog # A21247; Invitrogen, Carlsbad, CA, USA) for 1 h at room temperature in the dark. Finally, nuclei were stained with DAPI (Bioworld Technology CO., Ltd., Nanjing, China) and observed under an FV3000 Laser Scanning Confocal Microscope (Olympus Corporation, Tokyo, Japan).

## MSCs cocultured with macrophages

### Indirect coculture

MSCs were seeded in the upper compartment of Transwells (0.4-µm pore size, Costar, Corning, Tewksbury, MA) in 24-well plates. LPMs were collected and cocultured with MSCs in the lower compartment of Transwells. LPMs were coincubated in the presence of LPS (100 ng/mL) for 24 h. The supernatants were collected for ELISAs, and LPMs were collected for qRT-PCR.

### Direct coculture

LPMs were collected and cocultured with MSCs (MSCs: LPMs = 1:5) in the presence of LPS (100 ng/mL) for 24 h in standard 24-well plates. The supernatants were collected for ELISAs, and cells were collected for qRT-PCR.

### Cell migration

MSCs were seeded in the lower compartment of Transwells (3-µm pore size, Costar, Corning, Tewksbury, MA) in 24-well plates. LPMs were seeded in the upper chamber in the presence of LPS (100 ng/mL) for 24 h. After removal of the culture solution in the upper chamber and three washes with PBS, the upper chamber was fixed with 4% paraformaldehyde for 15 min and three washes with PBS. Then, ammonium oxalate crystal violet was added to the upper chamber and dyed for 15 min, the dye liquor was recovered, the dye liquor was slowly washed away with PBS. Cotton swab was used in the upper chamber to absorb water. The chamber was placed on a slide glass, images were taken and quantification was performed.

### Animals and experimental protocol

Healthy female Balb/c mice (8–10 weeks old) were obtained from Jiangsu Huachuang Xinnuo Pharmaceutical Technology Co., Ltd. (Taizhou, China), and they were housed under a standard 12 h/12 h light/ dark cycle. Mice had ad libitum access to food and water. All procedures involving mice were performed in strict accordance with protocols based on the Institutional Guidelines for Animal Care and Use and approved by the Animal Care Committee at Nanjing University (No. IACUC-D2202077).

The IUA murine model was established as previously described [17], dual methods using uterine curettage and Lipopolysaccharides (LPS) injection. In Result 1, mice were randomly divided into 14 groups, sham (3 h, 6 h, 12 h, 1 day, 2 day, 4 day, and 7 day), in which mice received only an incision wound without any treatment to the endometrium, and IUA groups (3 h, 6 h, 12 h, 1 day, 2 day, 4 day, and 7 day), which received curettage of bilateral intact endometrial with an intrauterine injection of LPS (1 mg/ml). In Result 1, the mice were intraperitoneally injected with 100 µL of clophosome A clodronate liposomes (CLL) into the abdominal cavity, followed by IUA. Mice were sacrificed on Day 2. In result 5, MSCs (1 × 10^6^ cells in 200 µL of phosphate-buffered saline (PBS)) were intraperitoneally injected 4 days after IUA modelling. Mice were sacrificed on Day 7. In result 6, the mice were administered 100 µL of CLL on Day 3. After 24 h, these mice were randomly assigned to receive intraperitoneal injections of PBS or MSCs (1 × 10^6^ cells/mouse). Mice were sacrificed on Day 7.

### RNA isolation and quantitative real-time PCR

Total RNA was extracted from tissues or cells using TRIzol reagent (Vazyme Biotech, China), following the manufacturer’s instructions. 1 µg total RNA was reverse transcribed in a 20-µL reaction system using the HiScript ll Q RT SuperMix for qPCR (Catalogue # R222-01; Vazyme Biotech, China). Differences among the target gene expression levels were calculated by the ΔΔCt method and normalised to the level of β-actin. The primers used are listed in Supplementary Table 1.

### Enzyme-linked immunosorbent assay (ELISA)

The protein levels of TNF-α, IL-1β, and IL-6 (BioLegend, SanDiego, CA, USA) in mouse serum, the protein level of TGF-β1 (Catalogue # KE10005; Proteintech, Wuhan, China) and STC-1 (Catalogue # EK1404; Boster, Wuhan, China) in the cell culture supernatants were detected using ELISA kits according to the manufacturer’s protocol.

### Western blotting

Proteins in cells were extracted with RIPA lysis buffer (Beyotime Biotechnology). Protein concentrations were determined using the Bradford assay (Pierce Biotechnology, Waltham, MA, USA). An equal amount (30 mg) of the protein lysate was subjected to sodium dodecyl sulfate-polyacrylamide gel electrophoresis (SDS-PAGE), and immunoblotted with the antibodies against HK2 (Catalogue # 2867 S; Cell Signaling Technology, Danvers, MA, USA).

### Flow cytometry assay

CD11b (Catalog # 101,206; Biolegend, San Diego, CA, USA), F4/80 (Catalog # 17-4801-82; Invitrogen, Carlsbad, CA, USA), CD102 (Catalog # 742,107; BD Pharmingen, San Jose, CA, USA), MHCII (Catalog # 12-5321-82; eBioscience, San Diego, CA, USA), GPX4 (Catalog # NBP3-08253 V; Novus Littleton, CO, USA) were used for labeling cells. All flow cytometry data were acquired using the Beckman Coulter Cytoflex S (Beckman Coulter, CA, USA) or BD FACSCalibur cytometer (BD Biosciences, San Diego, CA, USA) and analyzed using FlowJo software (Treestar, Inc., San Carlos, CA, USA).

### Cell viability assay

Cell Counting Kit-8 (CCK-8) assay was used according to the manufacturer’s instructions (Catalog # CK04, Dojindo, Tokyo, Japan).

### Small interfering RNAs (siRNAs)

SiRNAs targeting HK2 and STC-1 were purchased from RiboBio (Guangzhou, China). Cells were transfected with 100 nM siRNA or nontargeting siRNA controls using RiboFECT CP Reagent (Ribobio, Guangzhou, China, Catalog No: C10511-05) according to the manufacturer’s instructions. SiRNA sequences were as follows: HK2: 5′-GCAACATCCTGATCGATTT-3′; STC-1: 5′-AGACCACTGTGCCCAAACA-3′. HK2 and STC-1 mRNA and protein expression levels were assessed by qRT-PCR and western blotting or ELISA, respectively.

### Statistical analysis

All values in the graphs are presented as the mean ± SD. Unpaired Student’s t-tests were used to analyze the statistical significance. For all statistical tests, significance was set at *p* < 0.05. Data analysis was performed using GraphPad Prism 8.

## Results

### Migration of large peritoneal macrophages to the injured uteri is vital for the clearance of ferroptotic monocytes/macrophages in uteri

**Fig. 1:**
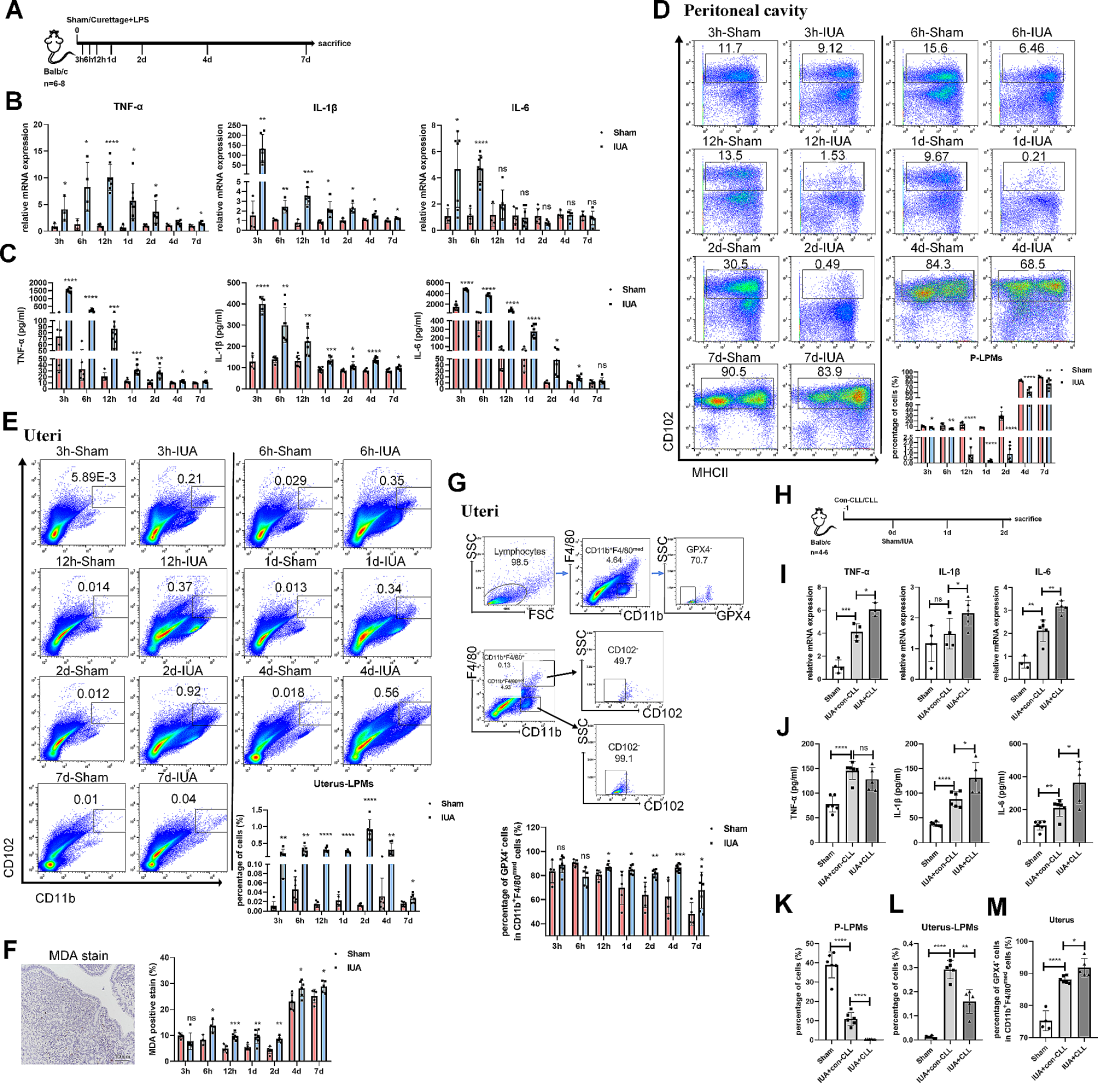
Accumulation of large peritoneal macrophages following injury in the uteri. (**A**) Schematic of in vivo IUA mice model setup (*n* = 6–8). (**B**) The mRNA expression levels of TNF-α, IL-1β, and IL-6 in the endometria of mice were determined by qRT-PCR (normalised to β-actin). (**C**) Serum concentrations of TNF-α, IL-1β, and IL-6 were measured by ELISA. (**D**) Flow cytometry analysis for LPMs isolated from peritoneal cavity harvested at different time points after injury. Cells were pregated on CD11b^+^. (**E**) Flow cytometry analysis for LPMs isolated from uteri harvested at different time points after injury. (**F**) Immunohistochemical staining of MDA in the endometria of mice (scale bar: 100 μm). (**G**) Flow cytometry analysis for GPX4 expression of CD11b^+^F4/80^med^ subsets in the uteri harvested at different time points after injury. (**H**) Schematic of in vivo LPMs depletion mice model setup (*n* = 6). (**I**) The mRNA expression levels of TNF-α, IL-1β, and IL-6 in the endometria of mice were determined by qRT-PCR (normalised to β-actin). (**J**) Serum concentrations of TNF-α, IL-1β, and IL-6 were measured by ELISA. (**K**) Flow cytometry analysis for LPMs isolated from peritoneal cavity harvested after 2-days injury. Cells were pregated on CD11b^+^. (**L**) Flow cytometry analysis for LPMs isolated from uteri harvested after 2-days injury. (**M**) Flow cytometry analysis for GPX4 expression of CD11b^+^F4/80^med^ subsets in the uteri harvested after 2-days injury. Values are mean ± SD. *p < 0.05, **p < 0.01, ***p < 0.001, ****p < 0.0001, ns denotes *p* > 0.05 (by unpaired Student’s *t* test)

Uterus is located in cavity and LPMs rapidly migrate into the region of injury/inflammation where they contribute to tissue repair. To investigate the dynamic changes of LPMs in vivo, we conducted a mouse model of IUA (Fig. 1A). In the sham group, the mice underwent laparotomy without any treatment of the endometrium. Mice were sacrificed on 3 h, 6 h, 12 h, day 1, day 2, day 4, or day 7 after the surgery. The expressions of TNF-α, IL-1β, and IL-6 in uteri and serum were significantly up-regulated in IUA groups (Fig. 1B, C). We evaluated the percentages of LPMs in the peritoneal cavity and uteri by flow cytometry. Compared with sham groups, the percentages of LPMs in the peritoneal cavity of IUA groups decreased significantly at every point of time (Fig. 1D). Meanwhile, the percentages of LPMs in the IUA uteri significantly increased (Fig. 1E). And the most notable changes occurred in day 2 both in the peritoneal cavity and uteri.

Malondialdehyde (MDA) staining revealed increased iron deposition and lipid peroxidation in the endometria of IUA mice (Fig. 1F), suggesting ferroptosis occurred in endometria of IUA uteri. To identify the main cell type undergoing ferroptosis, we used the flow cytometry to distinguish LPMs from monocytes/macrophages in the uteri, the results revealed the presence of two distinct macrophage populations within the uteri. CD102 has been identified as a specific marker for LPMs [25]. Nearly half of the macrophages expressed CD102 in the CD11b^+^F4/80^+^ population, while the CD102 expression were almost all negative within the CD11b^+^F4/80^med^ population, indicating that the LPMs were not present in CD11b^+^F4/80^med^ population, which might be monocytes/macrophages originally existed in the uteri (Fig. 1G). GPX4 is the only antioxidant enzyme that can directly reduce phospholipid peroxidation in cells and plays an important role in ferroptosis [26]. We found interestingly that after 12 h, GPX4-negative cells gated in CD11b^+^F4/80^med^ population significantly increased. These data indicated that monocytes/macrophages obviously underwent ferroptosis in the IUA uteri.

To detect whether LPMs that migrated to the uteri can engulf ferroptotic monocytes/macrophages, we injected intraperitoneally clodronate liposome (CLL) in advance to remove peritoneal macrophages (Fig. 1H). The expressions of TNF-α, IL-1β, and IL-6 in uteri and serum were significantly up-regulated in IUA + CLL group, compared with IUA + con-CLL group (Fig. 1I, J). Notedly, we found that when LPMs were depleted by CLL (Fig. 1K), the proportion of LPMs in the uteri decreased (Fig. 1L), while more importantly, the proportion of ferroptotic monocytes/macrophages in the uteri significantly increased (Fig. 1M). These results suggested that LPMs were responsible for the clearance of ferroptotic monocytes/macrophages in the injured endometria and inflammation resolution.

### LPM efferocytosis are verified in clearing ferroptotic monocytes/macrophages *in vitro*

**Fig. 2:**
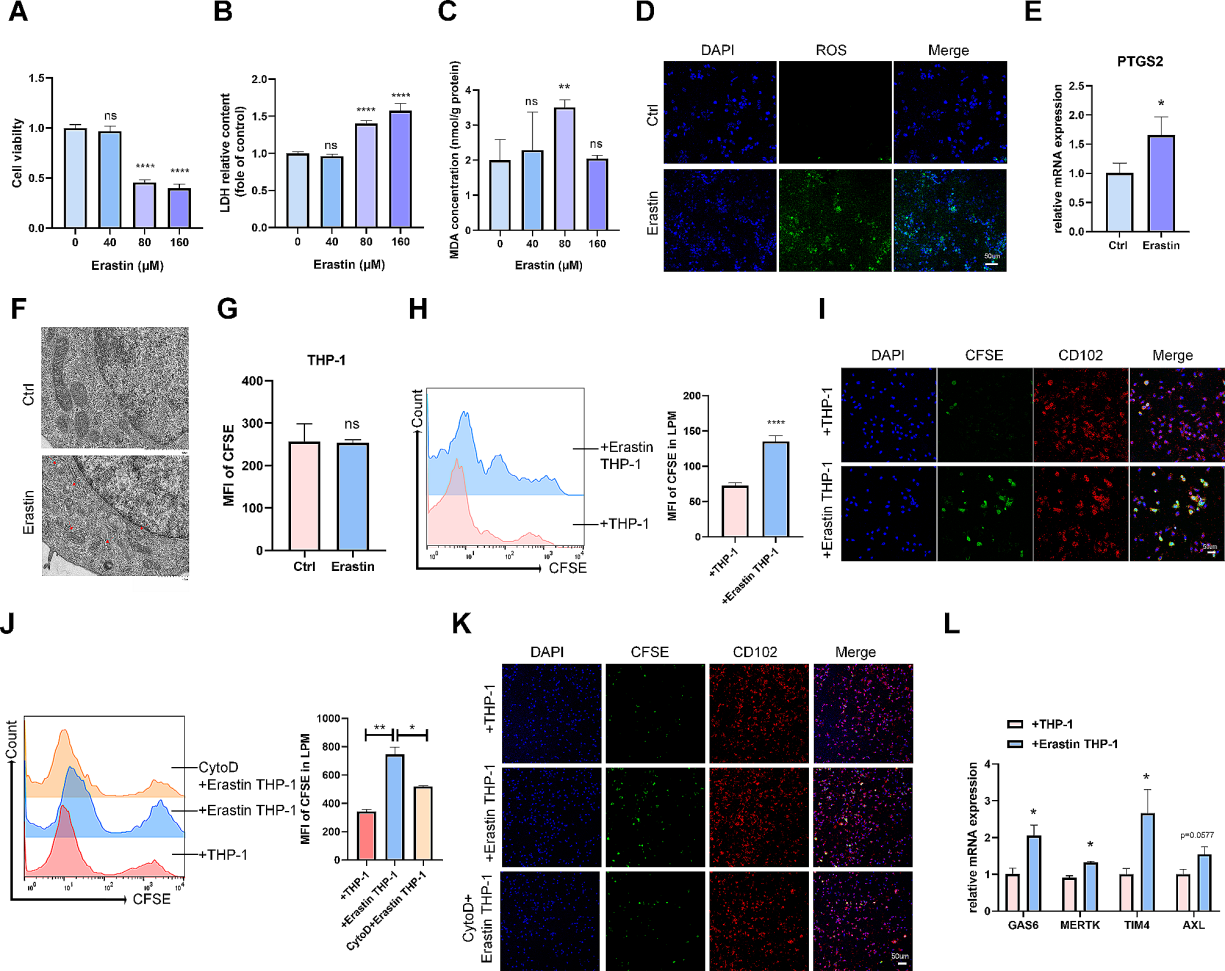
Ferroptotic THP-1 cells promote the efferocytosis of LPMs *in vitro*. (**A**) Cell viability, (**B**) LDH relative content, and (**C**) the MDA concentration in THP-1 cells stimulated with different concentrations of Erastin for 24 h. (**D**) The intracellular ROS level stimulated with 80 µM Erastin for 24 h was measured by confocal microscope. (scale bar: 50 μm) (**E**) qRT-PCR analysis showed the mRNA level of Ptgs2 in THP-1 cells stimulated with 80 µM Erastin for 24 h (normalised to β-actin). (**F**) Representative pictures acquired by transmission electron microscopy. Red arrows indicated mitochondrial cristae disappearance and outer membrane rupture. (**G**) THP-1 cells treated with or without Erastin for 24 h, then stained by CFSE. MFI of CFSE in THP-1 cells was detected by flow cytometry. LPMs were incubated with nontreated or Erastin-treated THP-1 cells stained by CFSE for 4 h. (**H**, **I**) Efferocytosis of LPMs from each group was determined by (**H**) flow cytometry and (**I**) confocal microscope (scale bar: 50 μm). (**J**, **K**) LPMs pretreated with 5 µM CytoD for 1 h were incubated with nontreated or Erastin-treated THP-1 cells stained by CFSE for 4 h. Efferocytosis of LPMs from each group was determined by (**J**) flow cytometry and (**K**) confocal microscope (scale bar: 50 μm). (**L**) LPMs were incubated with nontreated or Erastin-treated THP-1 cells for 4 h. The mRNA levels of efferocytosis related genes in LPMs were measured by qRT-PCR (normalised to β-actin). Values are mean ± SD. *p < 0.05, **p < 0.01, ****p < 0.0001, ns denotes *p* > 0.05 (by unpaired Student’s *t* test)

To induce ferroptosis in monocytes/macrophages, we stimulated the macrophage cell line THP-1 cells with Erastin and found that Erastin significantly downregulated the cell viability, upregulated the content of lactate dehydrogenase (LDH), MDA, ROS, and the mRNA expression of ferroptosis biomarker Ptgs2 in THP-1 cells (Fig. 2A-E). As ferroptosis coincided with morphologic changes, transmission electron microscopy studies revealed that Erastin induced mitochondrial cristae disappearance and outer membrane rupture in THP-1 cells (Fig. 2F).

Given the fact that LPMs possess the ability of efferocytosis to clear the dead cells. We hypothesized that LPMs engulf ferroptotic THP-1 cells. To examine this hypothesis, THP-1 cells were treated with or without Erastin. Expectedly, Erastin induction did not affect the basal level of 5(6)-carboxyfluorescein diacetate N-succinimidyl ester (CFSE) in THP-1 cells (Fig. 2G), then LPMs incubated with ferroptotic THP-1 cells pre-stained with CFSE. The MFI of CFSE dramatically increased in LPMs cocultured with Erastin-treated THP-1 cells, compared with LPMs cocultured with nontreated THP-1 cells (Fig. 2H, I). By contrast, Cytochalasin D (an efferocytosis inhibitor) treatment significantly reversed the MFI of CFSE in LPMs cocultured with Erastin-treated THP-1 cells (Fig. 2J, K). It was reported that macrophages can recognize and bind phosphatidylserine (PS) directly through transmembrane receptors such as TIM4 on the membrane surface. The recognition of PS by the tyrosine kinase receptor family (AXL, MERTK, TAM) on the membrane surface is mediated by secreting GAS6 and protein S as bridge molecules [27–29]. We found that the mRNA expression levels of GAS6, MERTK, TIM4, and AXL were upregulated in LPMs cocultured with Erastin-treated THP-1 cells, compared with LPMs cocultured with nontreated THP-1 cells (Fig. 2L).

Efferocytosis is thought to promote the pro-resolving phenotype by down-regulating proinflammatory cytokines and increasing pro-resolving factors [30]. We found that both mRNA and secretion levels of TGF-β1 were significantly upregulated, while the expressions of pro-inflammatory factors TNF-α, IL-1β, and IL-6 were significantly downregulated in LPMs cocultured with Erastin-treated THP-1 cells (Supplementary Fig. 1A-D). The efferocytosis-induced macrophage resolution pathways are reported to be triggered by activation of apoptosis cell (AC) receptors or molecules resulting from the phagolysosomal degradation of engulfed Acs [31, 32]. So we pre-treated LPMs with bafilomycin A1 (Baf A1), a lysosomal vacuolar ATPase inhibitor that blocks phagolysosomal AC degradation during efferocytosis. We found that Baf A1 treatment suppressed Erastin-treated THP-1 cells-induced increase in TGF-β1 and decrease in IL-1β (Supplementary Fig. 1E, F). All together, these studies exhibited that Erastin-induced ferroptotic THP-1 cells enhanced the efferocytic capability of LPMs and promoted LPMs skewing themselves toward alternative or repair phenotype.

### LPM efferocytosis against ferroptotic monocytes/macrophages depends on the PPARγ-HK2 pathway

**Fig. 3:**
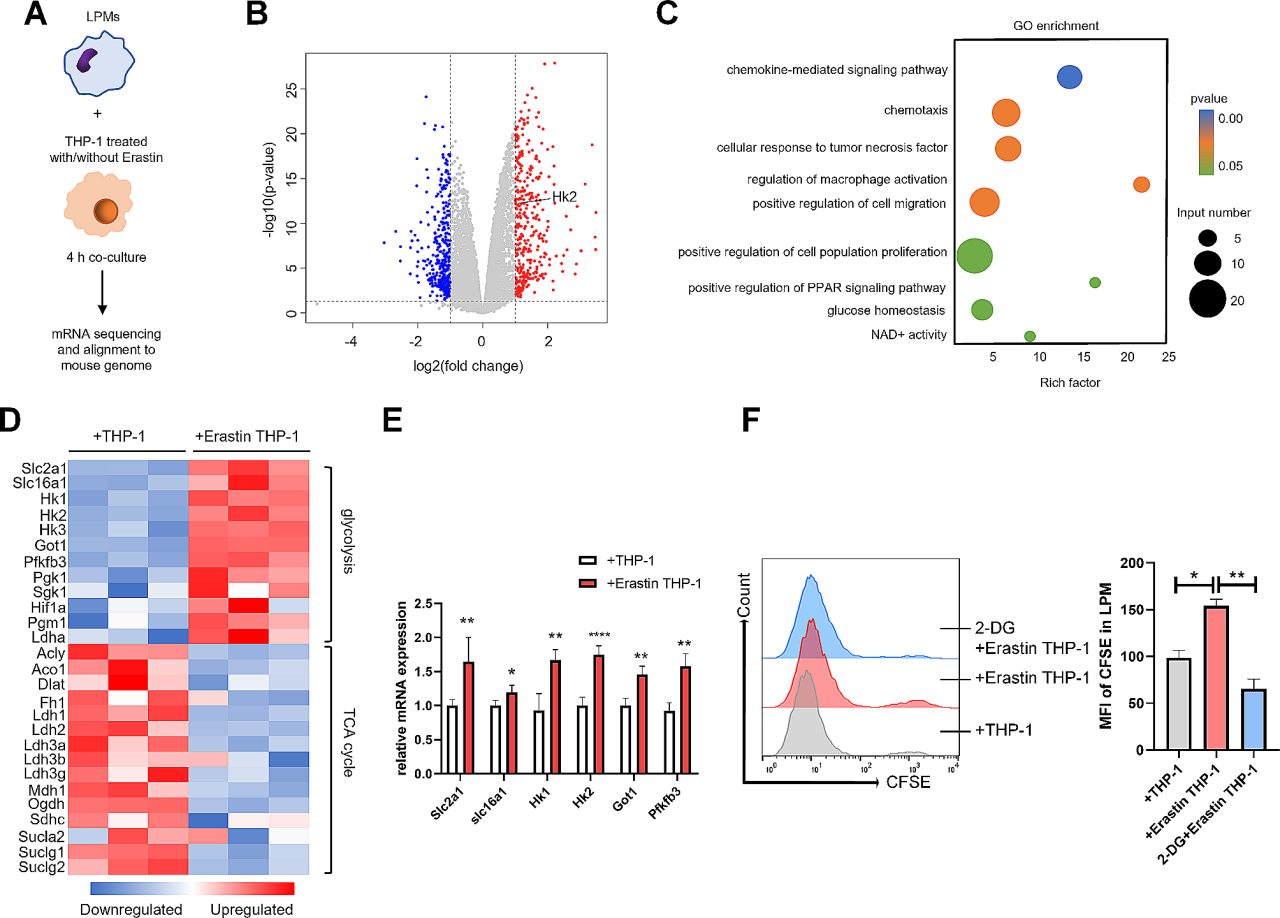
Transcriptional program is initiated during efferocytosis. (**A**) Schematic of in vitro coculture system setup. (**B**) The volcano figure showed fold-changes of genes in LPMs incubated with nontreated or Erastin-treated THP-1 cells. (**C**) GO enrichment analysis of all differentially expressed genes. (**D**) The thermogram of glycolysis and TCA cycle related genes expression pattern. (**E**) qRT-PCR analysis showed the glycolysis related genes in LPMs incubated with nontreated or Erastin-treated THP-1 cells for 4 h. LPMs pretreated with 10 mM 2-DG for 2 h were incubated with nontreated or Erastin-treated THP-1 cells stained by CFSE for 4 h (normalised to β-actin). (**F**) Efferocytosis of LPMs from each group was determined by flow cytometry. Values are mean ± SD. *p < 0.05, **p < 0.01, ****p < 0.0001 (by unpaired Student’s *t* test)

To identify the pathways potentially involved in efferocytosis, we performed RNA sequencing of LPMs engulfing ferroptotic or control THP-1 cells (Fig. 3A). Efferocytic LPMs displayed changes in multiple genes (Fig. 3B). Gene Ontology (GO) enrichment analysis showed that many signal transduction pathways including glucose homeostasis and PPAR signaling pathway were activated (Fig. 3C). Cluster heatmap revealed upregulation of multiple glycolysis genes, with concurrent downregulation of TCA cycle genes (Fig. 3D). We further verified these changes of glycolysis genes via qRT-PCR, and found that HK2 was the most varied gene (Fig. 3E). Further, LPMs pre-treated with 2-deoxyglucose (2-DG, a competitive inhibitor of hexokinase that block glycolysis) showed decreased efferocytosis (Fig. 3F).

**Fig. 4:**
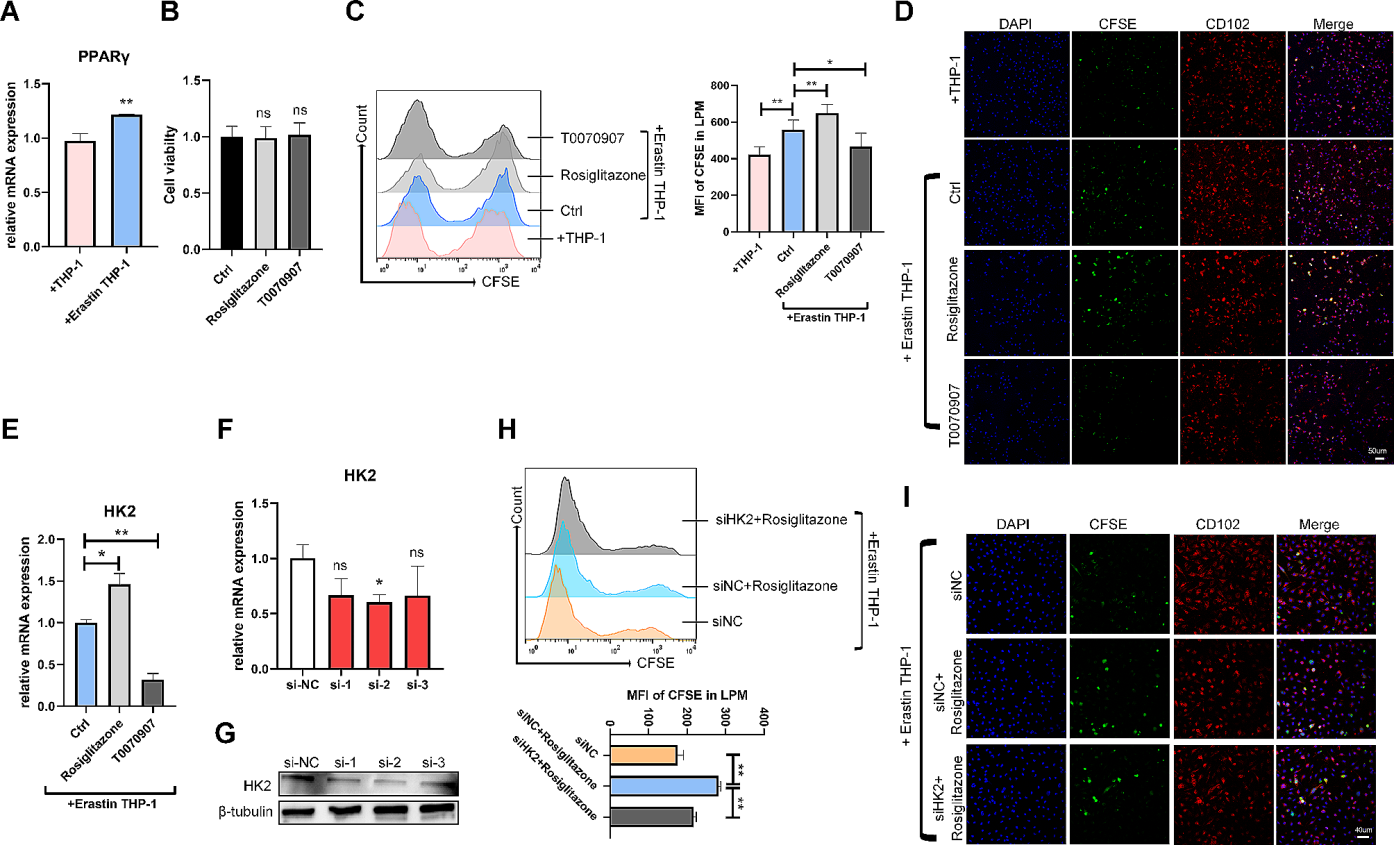
The PPARγ-HK2 pathway promotes the efferocytosis of LPMs against ferroptotic THP-1 cells. (**A**) The mRNA expression level of PPARγ in LPMs incubated with nontreated or Erastin-treated THP-1 cells for 4 h (normalised to β-actin). (**B**) Cell viability of LPMs treated with 10 µM PPARγ agonist, Rosiglitazone or 10 µM PPARγ inhibitor, T0070907 for 18 h. (**C**, **D**) LPMs treated as above were incubated with nontreated or Erastin-treated THP-1 cells for 4 h. Efferocytosis of LPMs from each group was determined by (**C**) flow cytometry and (**D**) confocal microscope (scale bar: 50 μm). (**E**) The mRNA expression level of HK2 in LPMs treated as above was detected by qRT-PCR. (**F**, **G**) qRT-PCR analysis (**F**) and Western blotting (**G**) showed the mRNA and protein levels of HK2 in LPMs transfected with three interfering fragments of HK2 (si-HK2) for 24 h. Full-length blots are presented in Supplementary Fig. 6. (**H**, **I**) LPMs were transfected with siHK2 for 24 h before stimulated with 10 µM Rosiglitazone, then incubated with Erastin-treated THP-1 cells stained by CFSE for 4 h. Efferocytosis of LPMs from each group was determined by (**H**) flow cytometry and (**I**) confocal microscope (scale bar: 40 μm). Values are mean ± SD. *p < 0.05, **p < 0.01, ns denotes *p* > 0.05 (by unpaired Student’s *t* test).

Peroxisome proliferator-activated receptor γ (PPARγ), a nuclear receptor activated by oxidized lipids, is a driver of macrophage metabolic programs, enhancing the ability to take up and digest dying cells [33]. As expected, the expression of PPARγ was upregulated in efferocytic LPMs cocultured with ferroptotic THP-1 cells (Fig. 4A). The efferocytosis of LPMs was upregulated by treatment with the PPARγ agonist, Rosiglitazone, while downregulated by treatment with the PPARγ inhibitor, T0070907. Rosiglitazone or T0070907 did not affect the cell viability of LPMs (Fig. 4B-D). Especially, the expression of HK2 in LPMs was upregulated by pretreatment with Rosiglitazone, while downregulated by T0070907 (Fig. 4E). To validate HK2 in LPM efferocytosis, we knocked down the expression of HK2 in LPMs by siRNA (Fig. 4F, G), and found that siHK2 reversed the efferocytosis of LPMs enhanced by Rosiglitazone (Fig. 4H, I). These results indicated LPMs effectively phagocytized to eliminate ferroptotic monocytes/macrophages via the PPARγ-HK2 pathway.

### MSCs promote the efferocytosis of LPMs to repair the injured uteri

**Fig. 5:**
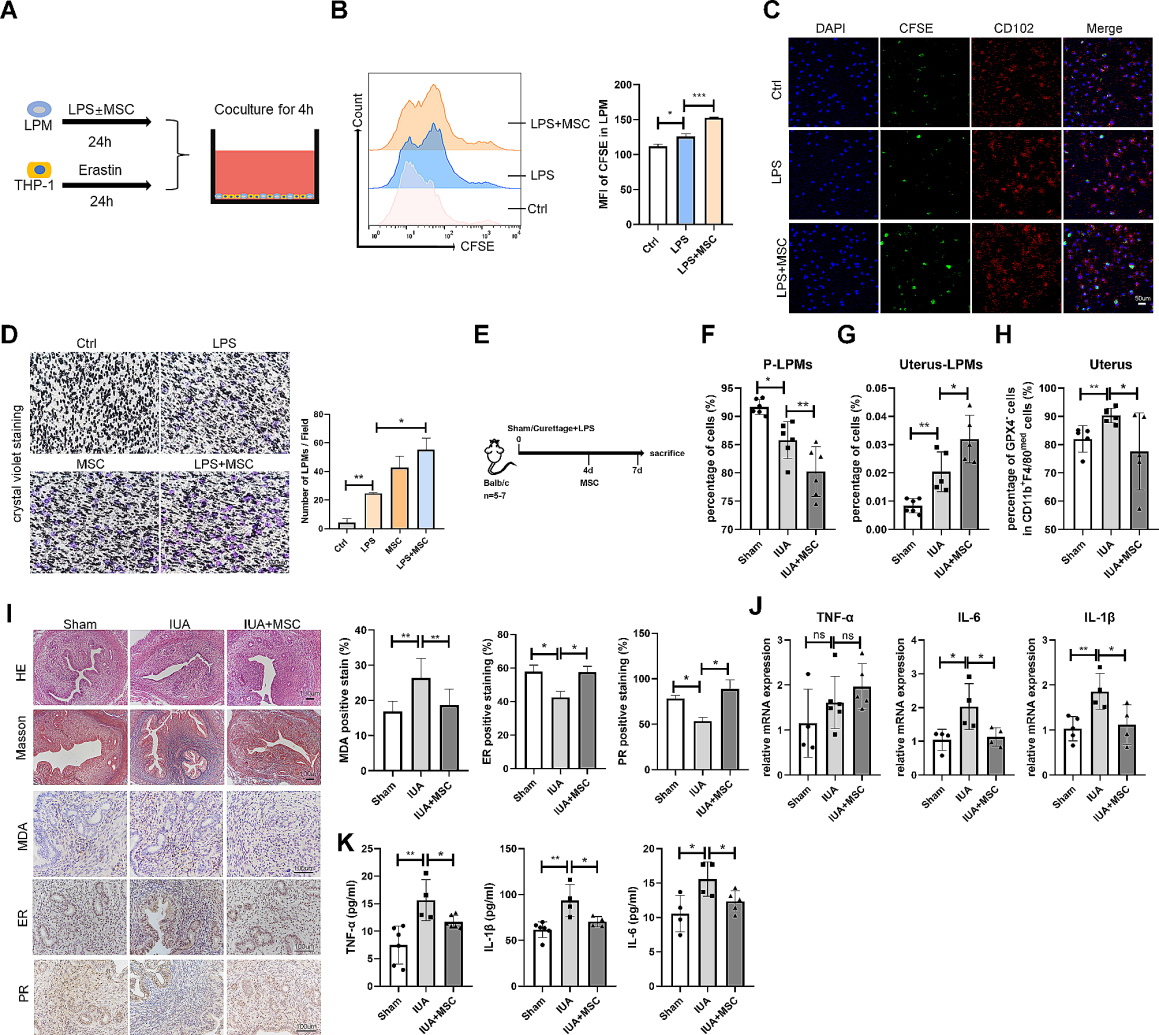
MSCs promote the efferocytosis and migration of LPMs to the injured uteri to repair the injured endometria. (**A**) Schematic of in vitro coculture system setup. (**B**, **C**) Efferocytosis of LPMs from each group was determined by (**B**) flow cytometry and (**C**) confocal microscope (scale bar: 50 μm). (**D**) Crystal violet staining of LPMs cocultured with MSCs or LPS (scale bar: 100 μm). (**E**) Schematic of in vivo experimental model design (*n* = 5–7). (**F**) Flow cytometry analysis for LPMs isolated from peritoneal cavity harvested after 7-days injury. Cells were pregated on CD11b^+^. (**G**) Flow cytometry analysis for LPMs isolated from uteri harvested after 7-days injury. (**H**) Flow cytometry analysis for GPX4 expression of CD11b^+^F4/80^med^ subsets in the uteri harvested after 7-days injury. (**I**) HE, Masson’s trichrome staining, MDA, ER, and PR stainings of endometrial tissues obtained from mice of three groups. Scale bar indicates 100 μm. (**J**) The mRNA expression levels of TNF-α, IL-1β, and IL-6 in the endometria of mice were determined by qRT-PCR (normalised to β-actin). (**K**) Serum concentrations of TNF-α, IL-1β, and IL-6 were measured by ELISA. Values are mean ± SD. *p < 0.05, **p < 0.01, ***p < 0.001, ns denotes *p* > 0.05 (by unpaired Student’s *t* test)

The cross-talk between MSCs and monocytes/macrophages is vital for the regulation of inflammation and repair, leading to the restoration of tissue homeostasis after injury. However, the effect of MSCs on LPMs needs to be unveiled. To mimic LPMs in the injured uteri, LPMs were pretreated with LPS, with or without MSCs for 24 h, then co-cultured with ferroptotic THP-1 cells (Fig. 5A). We found that MSCs significantly enhanced the efferocytosis of LPMs (Fig. 5B, C) and promoted polarization of LPMs to the M2 phenotype (Supplementary Fig. 2). The crystal violet assay showed that MSCs also promoted the migration ability of LPMs (Fig. 5D).

**Fig. 6:**
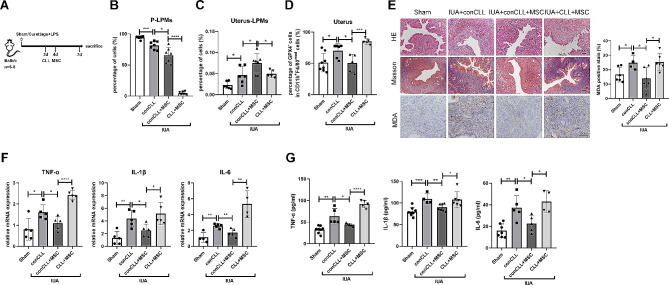
MSCs exert the effect of repairing the injured endometria through LPMs. (**A**) Schematic of in vivo experimental model design (*n* = 6–8). (**B**) Flow cytometry analysis for LPMs isolated from peritoneal cavity harvested after 7-days injury. Cells were pregated on CD11b^+^. (**C**) Flow cytometry analysis for LPMs isolated from uteri harvested after 7-days injury. (**D**) Flow cytometry analysis for GPX4 expression of CD11b^+^F4/80^med^ subsets in the uteri harvested after 7-days injury. (**E**) HE staining, Masson’s trichrome staining, and MDA staining of endometrial tissues obtained from mice of four groups. Scale bar indicates 100 μm. (**F**) The mRNA expression levels of TNF-α, IL-1β, and IL-6 in the endometria of mice were determined by qRT-PCR (normalised to β-actin). (**G**) Serum concentrations of TNF-α, IL-1β, and IL-6 were measured by ELISA. Values are mean ± SD. *p < 0.05, **p < 0.01, ***p < 0.001, ****p < 0.0001 (by unpaired Student’s *t* test)

Next, to detect the effects of MSCs on the regulation of the migration of LPMs and the therapeutic efficacy on the injured endometria in vivo, we established IUA mouse model (Fig. 5E). We found that MSCs significantly reduced the proportion of LPMs in the peritoneal cavity but increased the proportion of LPMs in the uteri. Deservedly, the proportion of ferroptotic monocytes/macrophages was reduced in the uteri (Fig. 5F-H). Importantly, MSCs drastically mitigated uterine pathological changes, including the increase of glands and the reduction of collagen deposition. MSCs also decreased the level of MDA in the endometrial stroma of IUA mice. Estrogen receptor (ER) and progesterone receptor (PR) are indicators of endometrial recovery in IUA [34]. The expressions of ER and PR were significantly increased in the MSCs-treated group compared with that in the IUA group (Fig. 5I). Meanwhile, MSCs reduced the expressions of inflammatory factors TNF-α, IL-1β, and IL-6 in the uteri and serum (Fig. 5J, K). To explore whether MSCs exerted their effects through LPMs, we injected intraperitoneally CLL to deplete LPMs before MSCs treatment (Fig. 6A) and found that the effects of MSCs were inhibited by LPMs depletion (Fig. 6B-E).

These data confirmed that MSCs alleviated endometrial injury through LPMs, which migrated into the uteri and exerted its efferocytosis against ferroptotic monocytes/macrophages in the injured uteri.

### STC-1 is necessary for MSCs to promote the efferocytosis of LPMs

**Fig. 7:**
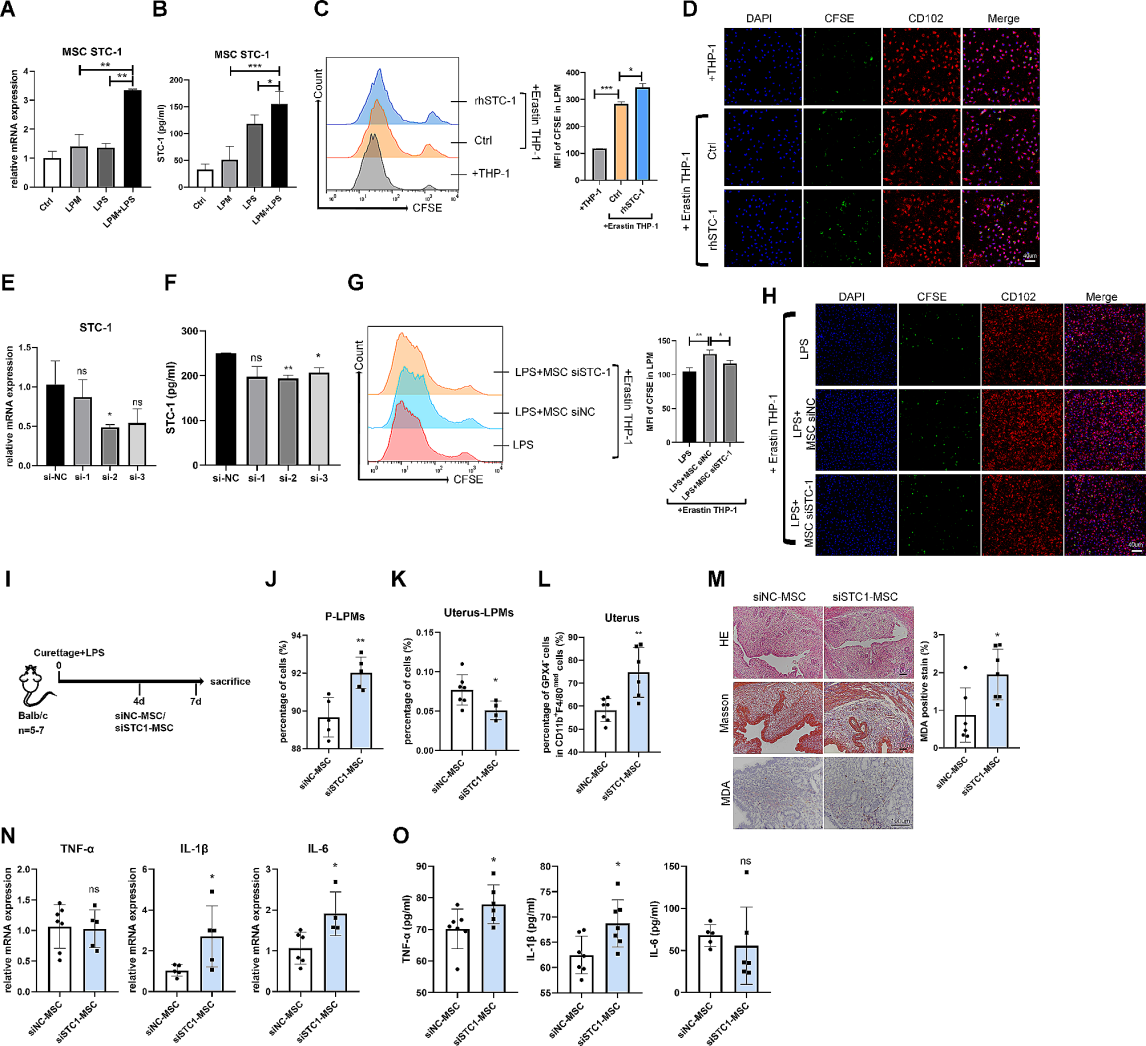
MSCs promote the efferocytosis of LPMs by secreting STC-1 *in vitro* and in *vivo*. (**A**, **B**) The mRNA (**A**) and protein (**B**) levels of STC-1 in MSCs cocultured with LPMs stimulated with LPS for 24 h were determined by qRT-PCR and ELISA (normalised to GAPDH). (**C**, **D**) LPMs were incubated with nontreated or Erastin-treated THP-1 cells in the presence of rhSTC-1 protein (100 ng/mL) for 4 h. Efferocytosis of LPMs from each group was determined by (**C**) flow cytometry and (**D**) confocal microscope (scale bar: 40 μm). (**E**, **F**) qRT-PCR analysis (**E**) and ELISA (**F**) showed the mRNA and protein levels of STC-1 in MSCs transfected with three interfering fragments of STC-1 (si-STC-1) for 24 h (normalised to GAPDH). (**G**, **H**) MSCs were transfected with siSTC-1 for 24 h before cocultured with LPMs, then LPMs were incubated with Erastin-treated THP-1 cells stained by CFSE for 4 h. Efferocytosis of LPMs from each group was determined by (**G**) flow cytometry and (**H**) confocal microscope (scale bar: 40 μm). (**I**) Schematic of in vivo experimental model design (*n* = 5–7). (**J**) Flow cytometry analysis for LPMs isolated from peritoneal cavity harvested after 7-days injury. Cells were pregated on CD11b^+^. (**K**) Flow cytometry analysis for LPMs isolated from uteri harvested after 7-days injury. (**L**) Flow cytometry analysis for GPX4 expression of CD11b^+^F4/80^med^ subsets in the uteri harvested after 7-days injury. (**M**) HE staining, Masson’s trichrome staining, and MDA staining of endometrial tissues obtained from mice of four groups. Scale bar indicates 100 μm. (**N**) The mRNA expression levels of TNF-α, IL-1β, and IL-6 in the endometria of mice were determined by qRT-PCR (normalised to β-actin). (**O**) Serum concentrations of TNF-α, IL-1β, and IL-6 were measured by ELISA. Values are mean ± SD. *p < 0.05, **p < 0.01, ***p < 0.001, ns denotes *p* > 0.05 (by unpaired Student’s *t* test)

To search for MSC-derived factors affecting the efferocytosis of LPMs, we detected MSCs-secreted prominent molecules that affect the macrophage phenotype, including stanniocalcin-1 (STC-1), hepatocyte growth factor (HGF), tumor necrosis factor (TNF)-stimulated gene and protein 6 (TSG-6), vascular endothelial growth factor (VEGF), and transforming growth factor-β1 (TGF-β1). When MSCs cocultured with LPS-stimulated LPMs relative to LPS or LPMs alone, the mRNA and protein levels of STC-1 were just upregulated in MSCs (Fig. 7A, B), while the changes of HGF, TSG-6, VEGF, and TGF-β1 were not impressive (Supplementary Fig. 3). Moreover, rhSTC-1 significantly enhanced the efferocytosis of LPMs (Fig. 7C, D). Contrarily, when STC-1 was knocked down in MSCs by siRNA (Fig. 7E, F), siSTC-1 could reverse the MSCs-enhanced efferocytosis of LPMs (Fig. 7G, H). Furthermore, in vivo studies revealed that STC-1 deficiency in MSCs also reversed the effect of MSCs on LPM efferocytosis as well as alleviation of endometrial fibrosis (Fig. 7I-O). These results revealed that the promotion of MSCs on LPM efferocytosis was in an STC-1-dependent manner.

## Discussion

Mature LPMs reside in the peritoneum with a less alternatively activated state, but once activated by signals derived from injury, they can be rapidly induced to express molecules associated with an alternative activated phenotype, which are thought to have tissue repair properties, showing decreased production of pro-inflammatory cytokines and increased production of immunosuppressive cytokines [9, 35]. Our results suggested that Erastin-induced ferroptotic THP-1 cells enhanced LPM efferocytosis and promoted LPMs skewing themselves toward alternative or repair phenotype, since the level of pro-resolving factor-TGF-β1 was significantly upregulated, while the expressions of pro-inflammatory factors TNF-α, IL-1β, and IL-6 were significantly downregulated in LPMs cocultured with Erastin-treated THP-1 cells (Supplementary Fig. 1A-D). Early activation of alternative activated LPMs may accelerate the end of inflammation and guarantee a rapid repair to preserve tissue integrity. Meanwhile, in the peritoneal cavity, LPMs also skew their phenotype toward alternative or repair macrophages, increasing their expression of arginase 1, decreasing their expressions of TNF-α, IL-1β, and IL-6 since 3 h after injury (supplementary Fig. 4), which may be attributed to the opening of the abdominal cavity and LPMs may also repair injury in the abdominal cavity.

LPMs process the ability to phagocytose apoptotic or necrotic cells [36, 37]. The clearance of these dead cells appeared to be vital for revascularization and restoration of normal tissue. Indeed, in our study, the proportion of ferroptotic monocytes/macrophages in the uteri was increased and the inflammation was aggravated once LPMs were depleted (Fig. 1G-M). Administration of CLL greatly reduced the proportion of LPMs in the peritoneal cavity but did not affect the proportion of ferroptotic monocytes/macrophages in the endometria. In addition, there was a decrease in the proportion of macrophages in abdominal organs such as spleen, while there were no significant differences in the proportion of macrophages in bone marrow and liver (supplementary Fig. 5). In vitro, Erastin-induced ferroptotic THP-1 cells enhanced the efferocytosis of LPMs (Fig. 2H-I).

It is generally believed that efferocytic macrophages are more M2-like [38], M2-like macrophages are reported to be OXPHOS-dependent, but Morioka S et al. reported that efferocytosis induced a metabolic gene program promoting glucose uptake and subsequent glycolysis, with downregulation of genes related to OXPHOS and FAO in the first few hours. Although glycolysis is linked to inflammation, they found that efferocytic phagocytes can affect non-engulfing naive macrophages towards anti-inflammatory polarization by releasing factors such as TGFβ and IL-10 during efferocytosis [39]. In consistent with this study, our work revealed that efferocytic LPMs engulfing ferroptotic THP-1 cells displayed changes in multiple genes (Fig. 3B), promoting multiple glycolysis genes, with concurrent downregulation of TCA cycle genes (Fig. 3D). Meanwhile, efferocytosis resulted in pro-repair phenotype of LPMs, with an induction of TGF-β1 and reduction of pro-inflammation factors TNF-α, IL-1β, and IL-6 (supplementary Fig. 1).

Like other tissue resident macrophages, LPMs perform an important immune surveillance function, maintaining healthy tissues by removing dead and dying cells and toxic materials. But LPMs also migrate to neighboring viscera in the peritoneal cavity such as liver and colon and help repairing damaged tissues [7, 40–42]. Tissue resident macrophages such as alveolar macrophages, Kupffer cells, microglia, osteoclasts, and LPMs all have specialized functions and phenotypes, local tissue-derived signals control the development of tissue-specific phenotypes [43, 44]. Studies reported that zinc finger transcription factor GATA6 uniquely expresses in LPMs, but not other macrophage subsets, regulates a tissue-specific gene expression program in LPMs, controls a subset of LPMs functions, including controlling anatomical localization of LPMs and IgA production by B-1 cells [7]. B-1 cells are a subtype of B cells, distributed uniquely in the peritoneal cavity and almost absent in lymphoid tissues. B-1 cells in peritoneal cavity continuously migrate to intestinal lamina propria, where they give rise to IgA-secreting cells [45, 46]. GATA6 in LPMs regulates gut IgA response mediated by peritoneal B-1 cells.

The cross-talk between MSCs and macrophages is vital for the regulation of inflammation and repair, leading to the restoration of tissue homeostasis after injury [47]. To comprehensively understand how MSCs affect LPMs upon injury, we analyzed the effects of MSCs on polarization, migration, and efferocytosis of LPMs under stress by using a coculture system of LPMs activated with LPS. We found that MSCs instructed LPMs to fulfill homeostatic functions through promoting anti-inflammatory polarization and migration into the injured uteri, and enhancing the efferocytosis of LPMs (Fig. 5).

The knowledge of regulatory factors from MSCs on the efferocytosis of LPMs was limited until now. There are several studies reported the substances of MSCs on the regulation of macrophage efferocytosis. Gómez-Ferrer M et al. found that extracellular vesicles derived from MSCs significantly improved the ability of monocyte-derived M1 macrophages to clear apoptotic neutrophils [48]. Zhang M et al. reported that exosomal miR‑16 and miR‑21 derived from bone marrow MSCs primed macrophages isolated from MRL‑*lpr* mice kidney for high efferocytosis activity, anti-inflammatory cytokines secretion, and Treg cell recruitment/differentiation [49]. Di Carlo SE et al. demonstrated that metabolically altered ADAM12^+^MSCs induced pathological angiogenesis and immunosuppression by promoting efferocytosis and polarization of BMDMs through overexpression of genes such as *Gas6*, *Lgals3* and *Csf1* [50]. Li LL et al. reported that adipose-derived MSCs reduced the activation of NLRC4 inflammasome in THP-1 cells induced by pseudomonas aeruginosa infection through the secretion of STC-1, thereby increasing the phagocytic ability of macrophages, and ultimately improving lung tissue damage [51]. A high-through assaying to identify regulatory factors from MSCs on the efferocytosis of LPMs will be useful and help to find the factor with the most significant regulatory effect, which will be carried out in our future studies.

STC-1 is a glycoprotein hormone expressed in various organs and tissues of the human body [52]. Studies have shown that STC-1 promotes cell proliferation and angiogenesis, reduces oxidative stress, and inhibits inflammation [53, 54]. It has been reported that STC-1 plays an important role in the immune regulation of MSCs [51, 55–58]. MSCs-derived STC-1 was required and sufficient for reduction of apoptosis of lung cancer epithelial cells [55]. STC-1 secreted by human umbilical MSCs can promote the secretion of IL-10 in alveolar macrophages, thus alleviating the inflammatory storm caused by acute respiratory distress syndrome [56]. Adipose-derived MSCs reduced the activation of NLRC4 inflammasome in macrophages induced by pseudomonas aeruginosa infection through the secretion of STC-1, thereby increasing the phagocytic ability of macrophages, and ultimately improving lung tissue damage [51]. In the present study, we found that MSCs expressed high levels of STC-1 when co-cultured with LPMs with LPS activation. STC-1 knockdown experiments showed that STC-1 deficiency in MSCs reversed the effect of MSCs on the regulation of LPMs and alleviation of endometrial fibrosis (Fig. 7I-O), which implied that STC-1 was an effector molecule of MSCs.

LPMs exert multiple functions after injury through efferocytosis, anti-inflammatory polarization, and suppression of other immune cells. As described in the present study, we revealed that LPMs were directed by MSCs to the injured endometria, so that to achieve homeostatic functions, promote the clearance of endometrial ferroptotic monocytes/macrophages and be vital effectors in inflammation and resolution.

## Conclusion

In summary, we demonstrated that LPMs migrated into the injured endometria to eliminate ferroptotic monocytes/macrophages. LPM efferocytosis can be promoted by MSCs-secreted STC-1. Collectively, our research demonstrated the essential functions of LPMs for endometrial repair and provided a novel mechanism of MSCs in repairing the injured endometria.

### Electronic supplementary material

Below is the link to the electronic supplementary material.

Supplementary Material 1

## Data Availability

All data generated or analyzed during study are included in the article.

## Abbreviations

IUA: Intrauterine adhesions
LPMs: Large peritoneal macrophages
MSCs: Mesenchymal stromal cells
CLL: Clodronate liposomes
PBS: Phosphate-buffered saline
LPS: Lipopolysaccharides
GO: Gene Ontology
MDA: Malondialdehyde
LDH: Lactate dehydrogenase
CFSE: 5(6)-carboxyfluorescein diacetate N-succinimidyl ester
PS: Phosphatidylserine
AC: Apoptosis cell
Baf A1: Bafilomycin A1
PPARγ: Peroxisome proliferator-activated receptorγ
2-DG: 2-deoxyglucose
ER: Estrogen receptor
PR: Progesterone receptor
STC-1: Stanniocalcin-1
HGF: Hepatocyte growth factor
TSG-6: Tumor necrosis factor
TNF: stimulated gene and protein 6
VEGF: Vascular endothelial growth factor
TGF-β1: Transforming growth factor-β1
