# Supplementary material for: MSCs promote the efferocytosis of large peritoneal macrophages to eliminate ferroptotic monocytes/macrophages in the injured endometria
Source: Stem Cell Res Ther. 2024 May 1;15:127. doi: 10.1186/s13287-024-03742-z (PMC11064342; doi:10.1186/s13287-024-03742-z)
Supplement: Supplementary file 1 — Supplementary Material 1 [file 13287_2024_3742_MOESM1_ESM.docx]

**Supplementary figures**


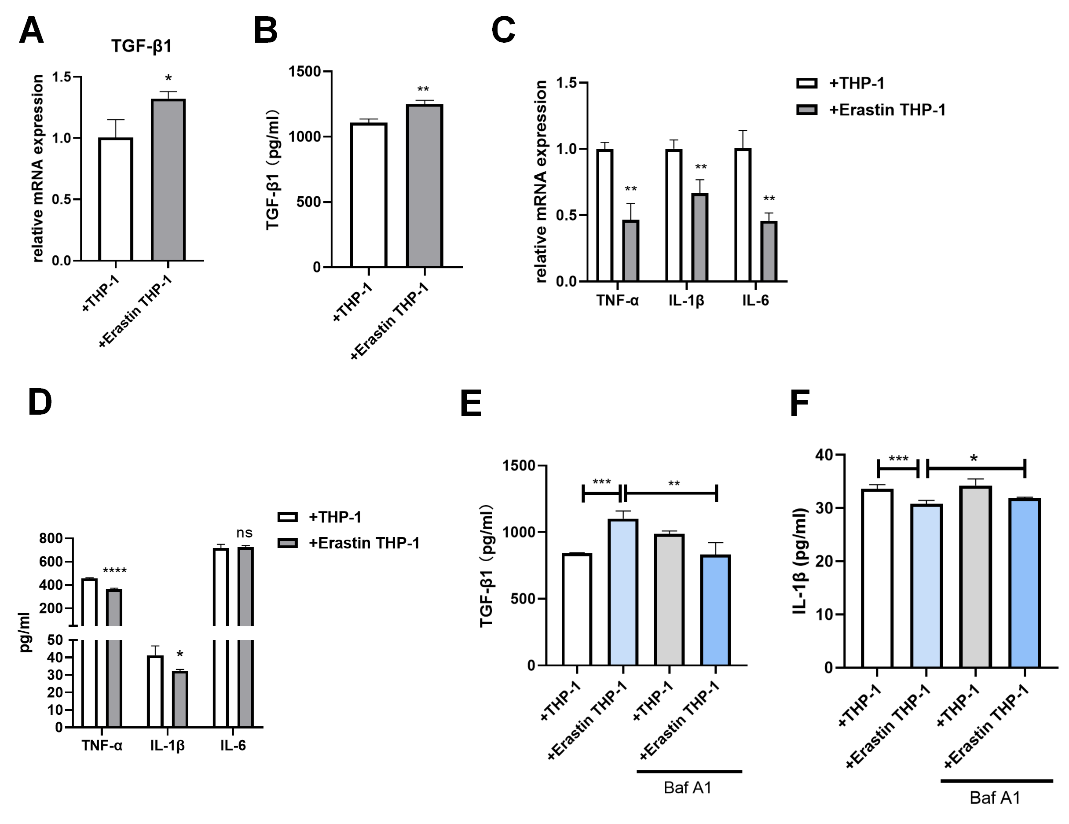


**Supplementary figure 1** (A, B) The mRNA (A) and protein (B) levels of TGF-β1 in LPMs incubated with nontreated or Erastin-treated THP-1 cells stained by CFSE for 4 h were determined by qRT-PCR and ELISA. (C, D) The mRNA (C) and protein (D) levels of TNF-α, IL-1β, and IL-6 in LPMs incubated with nontreated or Erastin-treated THP-1 cells stained by CFSE for 4 h were determined by qRT-PCR and ELISA. (E, F) LPMs pretreated with 1 nM BafA1 for 2 h were incubated with nontreated or Erastin-treated THP-1 cells for 4 h. The protein levels of TGF-β1 (E) and IL-1β (F) in LPMs were determined by ELISA. Values are mean ± SD. *p < 0.05, **p < 0.01, ***p < 0.001, ****p < 0.0001, ns denotes p > 0.05 (by unpaired Student’s *t* test).


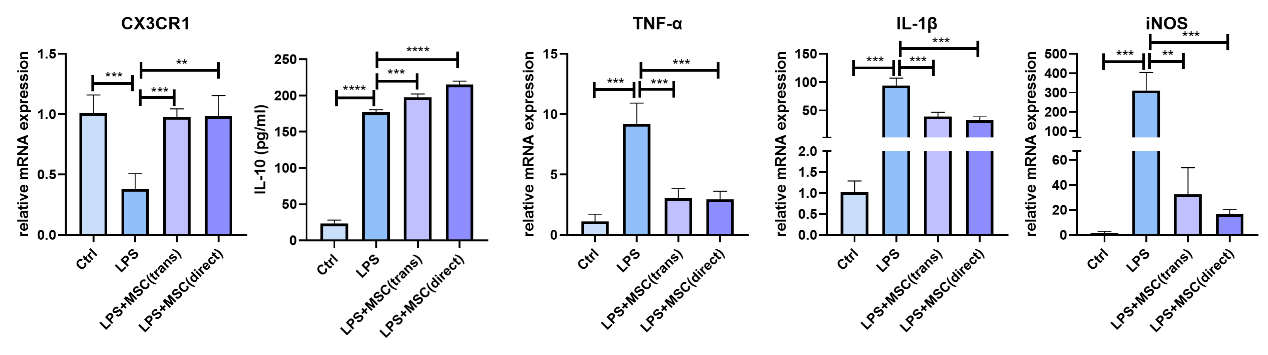


**Supplementary figure 2** The mRNA and protein levels of M1 and M2 phenotype genes in LPMs after direct coculture or indirect coculture with MSCs. Values are mean ± SD. **p < 0.01, ***p < 0.001, ****p < 0.0001 (by unpaired Student’s *t* test).


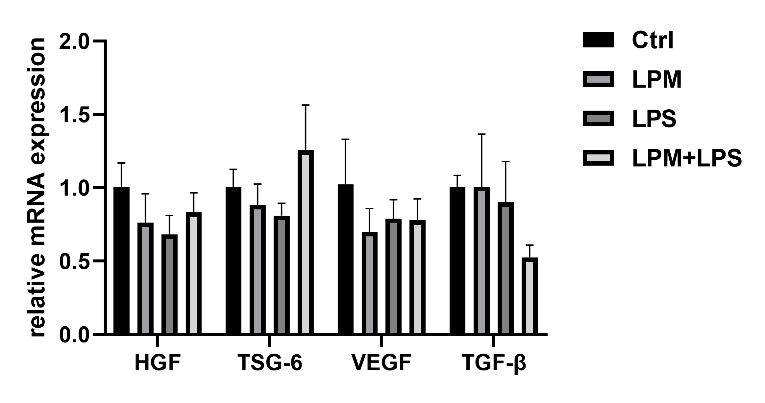


**Supplementary figure 3** The mRNA levels of HGF, TSG-6, AREG, and TGF-β1 in MSCs co-cultured with LPMs stimulated with or without LPS for 24 h were determined by qRT-PCR.


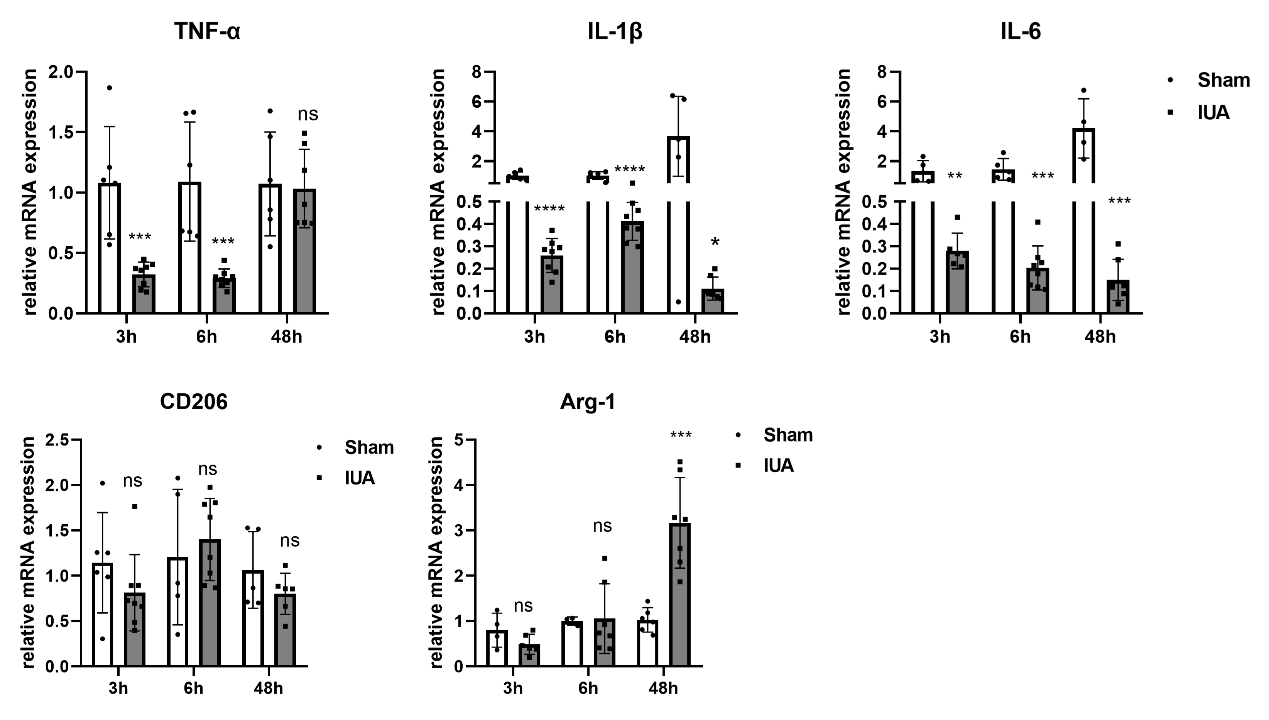


**Supplementary figure 4** The mRNA expression levels of pro-inflammation factors (TNF-α, IL-1β, and IL-6) and anti-inflammation factors (CD206 and Arg-1) in LPMs isolated from sham and IUA mice at different time points after injury were determined by qRT-PCR (normalised to β-actin). Values are mean ± SD. *p < 0.05, **p < 0.01, ***p < 0.001, ****p < 0.0001, ns denotes p > 0.05 (by unpaired Student’s *t* test).


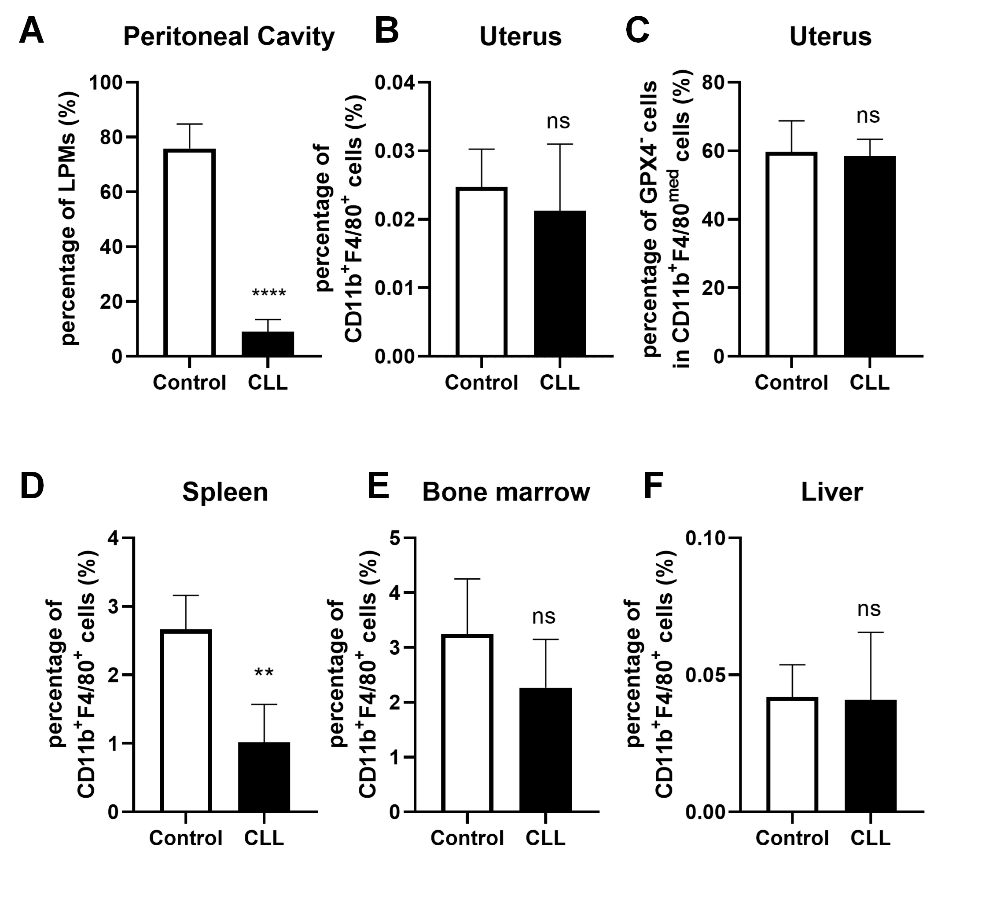


**Supplementary figure 5** Flow cytometry analysis in various intra-abdominal tissues after 3-days of CLL injection.


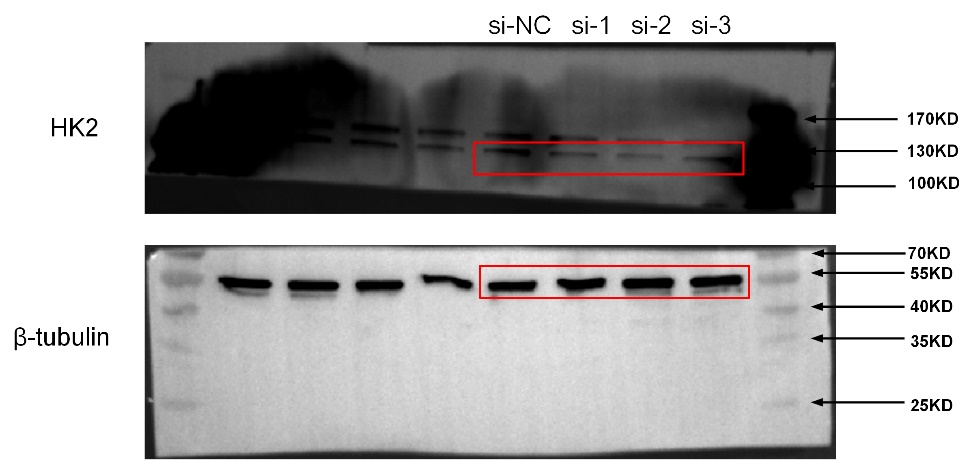


**Supplementary figure 6** Full-length blots of Figure 4G

**Table 1 Primers used in this study**

| **Gene** | Forward | Reverse |
| --- | --- | --- |
| Mm-TNF-α | GACGTGGAACTGGCAGAAGAG | TTGGTGGTTTGTGAGTGTGAG |
| Mm-IL-1β | GCAACTGTTCCTGAACTCAACT | ATCTTTTGGGGTCCGTCAACT |
| Mm-iNOS | GTTCTCAGCCCAACAATACAAGA | GTGGACGGGTCGATGTCAC |
| Mm-IL-6 | TAGTCCTTCCTACCCCAATTTCC | TTGGTCCTTAGCCACTCCTTC |
| Mm-CX3CR1 | GAGTATGACGATTCTGCTGAGG | CAGACCGAACGTGAAGACGAG |
| Mm-TGF-β1 | CTCCCGTGGCTTCTAGTGC | GCCTTAGTTTGGACAGGATCTG |
| Mm-β-actin | AGGTGACAGCATTGCTTCTG | GGGAGACCAAAGCCTTCATA |
| Hs-AREG | GTGGTGCTGTCGCTCTTGATA | CCCCAGAAAATGGTTCACGCT |
| Hs-HGF | GCTATCGGGGTAAAGACCTACA | CGTAGCGTACCTCTGGATTGC |
| Hs-TSG-6 | TCACATTTCAGCCACTGCTC | AGACCGTGCTTCTCTGTGGT |
| Hs-STC-1 | CAGCTGCCCAATCACTTCTC | TCTCCATCAGGCTGTCTCTGA |
| Hs-VEGF | AGGGCAGAATCATCACGAAGT | AGGGTCTCGATTGGATGGCA |
| Hs-GAPDH | GGAGCGAGATCCCTCCAAAAT | GGCTGTTGTCATACTTCTCATGG |
